# Criteria to assess potential reverse innovations: opportunities for shared learning between high- and low-income countries

**DOI:** 10.1186/s12992-016-0225-1

**Published:** 2017-01-25

**Authors:** Onil Bhattacharyya, Diane Wu, Kathryn Mossman, Leigh Hayden, Pavan Gill, Yu-Ling Cheng, Abdallah Daar, Dilip Soman, Christina Synowiec, Andrea Taylor, Joseph Wong, Max von Zedtwitz, Stanley Zlotkin, William Mitchell, Anita McGahan

**Affiliations:** 1 Department of Family and Community Medicine, University of Toronto, 500 University Ave, Toronto, Ontario M5G 1V7 Canada; 2 Women’s College Hospital, 76 Grenville St, Toronto, Ontario M5S 1B2 Canada; 3 North York General Hospital, 4001 Leslie St, Toronto, Ontario M2K 1E1 Canada; 4 Faculty of Medicine, University of Toronto, 1 King’s College Cir #3172, Toronto, Ontario M5S 1A8 Canada; 5 Department of Chemical Engineering & Applied Chemistry, Faculty of Applied Science & Engineering, University of Toronto, 200 College Street, Toronto, Ontario M5S 3E5 Canada; 6 Dalla Lana School of Public Health and Department of Surgery, University of Toronto, 155 College St., Toronto, Ontario M5T 1P8 Canada; 7 Rotman School of Management, University of Toronto, 105 St George St, Toronto, Ontario M5S 3E6 Canada; 8 Results for Development, 1514 17th St NW, Washington, DC 20036 USA; 9 Innovations in Healthcare, Duke University, 324 Blackwell Street, Suite 960, Durham, North Carolina 27701 USA; 10 Munk School of Global Affairs and Department of Political Science, University of Toronto, 1 Devonshire Place, Toronto, Ontario M5S 3K7 Canada; 11 Department of Strategic Management, Kaunas University of Technology, Gedimino 50 – 414, LT-44029 Kaunas, Lithuania; 12 Department of Paediatrics, Dalla Lana School of Public Health and Department of Nutritional Sciences, University of Toronto 555 University Avenue, Toronto M5G 1X8 Ontario, Canada; 13 Centre for Global Child Health and Research Institute, Hospital for Sick Children, 525 University Avenue, Suite 701, Toronto, Ontario M5G2L3 Canada

**Keywords:** Reverse innovation, Innovation, Healthcare delivery models, Low- and middle-income countries, Global health

## Abstract

**Background:**

Low- and middle-income countries (LMICs) are developing novel approaches to healthcare that may be relevant to high-income countries (HICs). These include products, services, organizational processes, or policies that improve access, cost, or efficiency of healthcare. However, given the challenge of replication, it is difficult to identify innovations that could be successfully adapted to high-income settings. We present a set of criteria for evaluating the potential impact of LMIC innovations in HIC settings.

**Methods:**

An initial framework was drafted based on a literature review, and revised iteratively by applying it to LMIC examples from the Center for Health Market Innovations (CHMI) program database. The resulting criteria were then reviewed using a modified Delphi process by the Reverse Innovation Working Group, consisting of 31 experts in medicine, engineering, management and political science, as well as representatives from industry and government, all with an expressed interest in reverse innovation.

**Results:**

The resulting 8 criteria are divided into two steps with a simple scoring system. First, innovations are assessed according to their success within the LMIC context according to metrics of improving accessibility, cost-effectiveness, scalability, and overall effectiveness. Next, they are scored for their potential for spread to HICs, according to their ability to address an HIC healthcare challenge, compatibility with infrastructure and regulatory requirements, degree of novelty, and degree of current collaboration with HICs. We use examples to illustrate where programs which appear initially promising may be unlikely to succeed in a HIC setting due to feasibility concerns.

**Conclusions:**

This study presents a framework for identifying reverse innovations that may be useful to policymakers and funding agencies interested in identifying novel approaches to addressing cost and access to care in HICs. We solicited expert feedback and consensus on an empirically-derived set of criteria to create a practical tool for funders that can be used directly and tested prospectively using current databases of LMIC programs.

## Background

There has always been an imperative to do more with less in low- and middle-income countries (LMICs) in general, but particularly in healthcare [1]. While investment in healthcare has been increasing for many of these countries, the needs and challenges faced by LMICs are greater than in high-income countries (HICs), and the resources are much more limited [2, 3].^1^ The ability of LMIC organizations to innovate is facilitated by the absence of constraining legacy systems, a flexible regulatory environment, low performance of standard approaches, and an urgency to do things better and cheaper [4]. As a result, many innovations that originate in LMICs have been shown to reduce cost, improve quality, and enhance access [5]. The relevance of these innovations to HIC settings has only recently been acknowledged, due to a growing pressure to control costs and improve access for marginalized groups [4, 6, 7]. This has increased interest in shared learning about innovations between low- and high-resource settings,[8] and the adaptation of LMIC innovations to HICs has been described as “reverse innovation” [4, 9]. While the use of the term “reverse” is controversial, the opportunities for HICs to learn from LMIC innovation are compelling. This novel approach to harness LMIC inventions in healthcare and life sciences could rapidly generate promising options for developed countries, overcoming some constraints to innovation in high-income settings.

The potential for LMIC innovations in HICs in medical-device and health product development, process-of-care improvements, and policy innovations, is starting to be realized. This is evidenced by such cases as General Electric India’s $800 electrocardiogram, now sold in 194 countries around the world [9]; the construction of Health City Cayman Islands, a 104-bed hospital using low-cost, high-throughput methods based on Narayana Hrudayalaya’s model in India [10]; adaptation of a community health worker model from Kenya to New York City by City Health Works [11, 12]; and implementation of the Opportunity NYC – Family Rewards program in New York, inspired by Mexico’s *Oportunidades* conditional cash transfer program [13]. Many of these initiatives have taken hold, and some have struggled; the initial Opportunity NYC program was discontinued, for instance, because of high cost and marginal effects [14].

While the impact of specific innovations has varied, their potential has garnered attention from a variety of stakeholders in HICs. They seek improvements involving efficiency in discovery [15], implementation of low-cost innovations to manage rising costs and a shrinking tax base, and reduction of barriers to moving products and services across national boundaries. Those interested in adapting LMIC innovations to HIC contexts include funders such as the Robert Wood Johnson Foundation and the Commonwealth Fund in the US [16, 17] and the Tekes Finnish Funding Agency for Innovation [18].

Despite the limited number of current examples, the pace of development of new products, processes, and policies from LMICs that are relevant to HICs is only going to increase, as disease patterns converge and healthcare coverage in low-resource settings increases [19]. With increased opportunities for funding and more LMIC-HIC partnerships, the capacity to develop and test new approaches from LMICs is rapidly growing [8]. However, replicating any innovation is challenging, and replicating in a very different context even more so; therefore, many factors must be considered in deciding to implement a new health model. Unsuccessful efforts such as the Opportunity NYC program underline such risks. The literature on reverse innovation describes examples and processes for how to develop reverse innovations [20], [9], with one article describing a competition for selecting LMIC innovations to test in HIC settings developed on an ad hoc basis [21]. This presents a critical opportunity for standardizing an approach to the evaluation of potential reverse innovations. To this end, we propose a screening mechanism to identify and promote those innovations from low- and middle-income markets with the greatest potential for application in HICs.

## Methods

Using an iterative, qualitative approach involving literature review, primary case study testing, secondary database review, and a modified Delphi process, we developed a two-part screening tool to identify innovations with the potential to improve care and/or reduce costs in HICs.

### Literature review and initial criteria development

Initial development of the tool began with review of the relevant literature around reverse innovation, using search terms such as “reverse innovation”, “frugal innovation”, “embedded innovation”, and “global health innovation”. From this, we developed a candidate set of 11 criteria to test further and refine. We then shared these initial criteria with six key informants in the global health field, including researchers, funders and clinicians based in HICs with extensive experience engaging with and assessing Canadian and LMIC health innovations. Based on their feedback, we revised the initial categories, resulting in eight refined criteria.

### Primary testing on case studies of innovative programs

Next, we applied the criteria to brief case studies we had developed for four LMIC health programs in the CHMI database [22] that our team had previously identified as having potential for adaptation in HICs. This allowed us to explore the relevance and utility of the eight criteria, and from this case study review, we developed a basic scoring system and divided the eight criteria into two steps:

- Evaluation of the program’s success within the LMIC context.
- Evaluation of the program’s likelihood to be a reverse innovation in an HIC.

### Secondary testing with expanded sample of programs

We then tested the criteria on 60 programs in the Center for Health Market Innovation database randomly sampled within five categories: innovative products, services, organizational structures, information and communication technologies, and financial models. Through this testing along with discussion and review of our findings, we developed a set of nine criteria with revised definitions and refined cut-off scores to identify likely and unlikely reverse innovations.

### Expert consultation in modified Delphi process

The resulting nine criteria were reviewed by a Reverse Innovation Working Group comprised of 31 experts in medicine, engineering, management, and political science, as well as representatives from government and industry, all with an expressed interest in reverse innovation. Many of the members were judges in a national reverse innovation competition held in Canada in 2014 [21]. Feedback was solicited using a modified Delphi process, an approach used to facilitate communication and build consensus amongst a group of experts on a particular topic [23]. This involved a first round of feedback with the group convening in a teleconference to provide open-ended feedback on the criteria. Afterwards, focused feedback requesting input on specific questions was solicited electronically from the working group. This feedback was used to revise the criteria, which were then shared again along with another set of focused questions that were discussed at an in-person meeting with the working group. Feedback from this group was again incorporated into the criteria, and these revised criteria, including the scoring system and suggested cut-offs scores, were shared with working group members electronically, at which point we achieved consensus.

## Results

The resulting tool consists of 8 criteria divided into 2 steps with a simple scoring system.

**Fig. 1:**
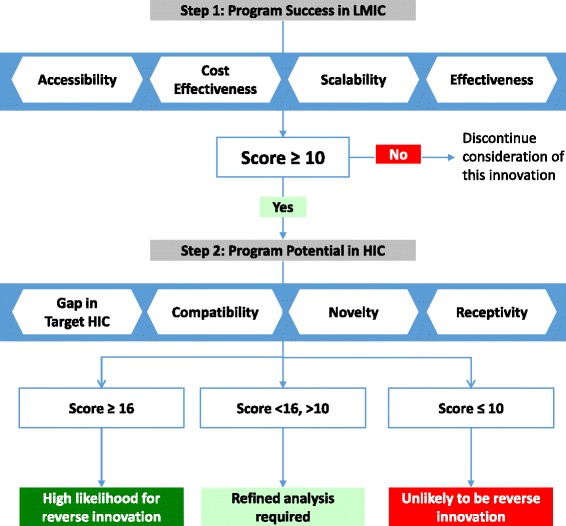
*Criteria Scoring System*. 0 No information exists, or the criterion is not applicable. 1 Demonstration that this has not been achieved. 2 Uncertain or conflicting demonstration. 3 Some demonstration of achievement. 4 Strong demonstration of achievement. 5 Significant demonstration of achievement. *Definitions of Individual Criteria*. Accessibility: Innovation increases access of products or services through increasing financial, geographic and/or social access. Cost Effectiveness: Innovation improves cost effectiveness to payer, provider, or end user. Scalability: Innovation increases scope, geographic cover, or customer base. Effectiveness: Documentation of effectiveness of innovation using appropriate evaluative methods. Gap in Target HIC: Creating solutions for unsolved (or imperfectly solved) challenges or unaddressed health issues or service gaps. Compatibility: Compatible with healthcare infrastructure in the target HIC country. Novelty: Innovation is a novel approach or an established innovation used in a new way that has great promise. Receptivity: Openness and engagement of partners as well as those not considered partners but who may be impacted by the innovation

Figure 1 presents the final scoring system, which consists of two parts: success in the LMIC and the potential for success in an HIC. The first part includes assessments of accessibility, cost effectiveness, scalability, and overall effectiveness in the LMIC. The total cut-off score of 10 is low to act as a coarse screen leading to the second phase. The second part reviews the health challenge addressed by the innovation, the compatibility with infrastructure and regulations, the novelty of the innovation, and the receptivity for the innovation in the HIC. The scores for this second part indicate whether the innovation is likely or unlikely to be a successful reverse innovation, or requires further investigation.

### Case examples

Tables 1 and 2 provide examples of application of the criteria to two different programs drawn from the CHMI database [22]. Canada is chosen as the target HIC for these examples, as Canada was the primary location for the majority of the authors.

**Table 1:** Applying the Reverse Innovation Criteria: Uganda Health Information Network (UHIN)

Brief description of initiative: *An information network for community health workers using low-cost PDAs and cellular networks to collect and share critical health data*			
**Step 1**			
**Criteria**	**Description**	**Score**	**Rationale**
1. Accessibility	Innovation increases access of products or services through increasing affordability, geographic access, and/or social access.	4	The program provides community health workers with PDAs that automatically load public health data to a centralized data bank. This helps with disease surveillance to ensure the right medicines and services are directed where needed.
2. Cost Effectiveness	Innovation improves cost effectiveness to payer, provider, or end user.	5	A study by independent consultants reported a savings of 25% per unit compared to traditional manual paper data collection.
3. Scalability	Innovation increases scope, geographic cover, or customer base.	4	There are 700 health workers in the program. There are 175 remote health facilities in the country that now have PDA capability, serving 1.5 million people.
4. Effectiveness	Documentation of effectiveness of innovation using appropriate evaluative methods	3	The cost-effectiveness of the program has been evaluated by independent consultants. The program is currently evaluating its health impact on healthcare planning, resource allocation, and delivery.
	TOTAL SCORE	16	Conclusion: Move to Step 2
**Step 2**			
**Criteria**	**Description**	**Score**	**Rationale**
5. Gap in Target HIC	Creating solutions for unsolved (or imperfectly solved) challenges or unaddressed health issues or service gaps.	5	Information integration and digitalization, with particular attention to cost control, are extremely important challenges for the Canadian health system.
6. Compatibility	Compatible with healthcare infrastructure in the target HIC.	3	It is unclear how this information system would be regulated in the Canadian context, particularly with privacy considerations. However, the system could likely be adapted to meet Canadian regulations.
7. Novelty	The innovation is a novel approach or an established innovation used in a new way that has great promise.	4	PDAs and smartphones remain a relatively innovative, uncommon tool for health data collection in Canada.
8. Receptivity	Openness and engagement of partners as well as those not considered partners but who may be impacted by the innovation.	4	The IDRC and former CIDA are the primary funders of this initiative, suggesting strong connections to Canada.
	TOTAL SCORE	16	Conclusion: Score is ≥16. Likely to be a reverse innovation.

**Table 2:** Applying the Reverse Innovation Criteria: Bloomberg Philanthropies Maternal Health Initiative (BPMHI)

Brief description: *The program trains assistant medical officers and midwives in remote areas to perform life-saving procedures including caesarean sections and upgrades isolated health centers*			
**Step 1**			
**Criteria**	**Description**	**Score**	**Rationale**
1. Accessibility	Innovation increases access of products or services through increasing affordability, geographic access, and/or social access.	5	The program has upgraded 9 remote health centers, increasing geographic access to services provided locally; prior to the program, patients had to travel 3-4 hours to the nearest hospital.
2. Cost Effectiveness	Innovation improves cost effectiveness to payer, provider, or end user.	4	Indirectly, the program has improved cost effectiveness by "up-training" midwives to perform more complex tasks such as c-sections, reducing the need for more costly healthcare providers.
3. Scalability	Innovation increases scope, geographic cover, or customer base.	4	Since inception, the program has increased its coverage from 9 sites to 12, and has been expanding to e-Learning platforms in 2013. Deliveries at all intervention hospitals have increased from 3,500 deliveries per year before the program to 9,000 deliveries per year after the program launch.
4. Effectiveness	Documentation of effectiveness of innovation using appropriate evaluative methods.	3	One district where the program operates experienced a 32% decline in maternal deaths after the program was implemented. However, information on evalulation techniques and reporting on impact for all sites is limited.
	TOTAL SCORE	16	Conclusion: Move to Step 2
**Step 2**			
**Criteria**	**Description**	**Score**	**Rationale**
5. Gap in Target HIC	Creating solutions for unsolved (or imperfectly solved) challenges or unaddressed health issues or service gaps.	4	Access to quality maternity care services, particularly in rural and northern areas, is an important healthcare issue in Canada.
6. Compatibility	Compatible with healthcare infrastructure in the target HIC.	1	Midwives and physician assistants play a limited role in Canada as of present. Implementation of such a program would challenging given current regulations and staffing levels.
7. Novelty	The innovation is a novel approach or an established innovation used in a new way that has great promise.	3	Midwifery and physician-assistant - performed c-sections are quite a novel concept; however refurbishing rural hospitals is not.
8. Receptivity	Openness and engagement of partners as well as those not considered partners but who may be impacted by the innovation.	1	This initiative does not involve Canadian partners or Canadian stakeholders.
	TOTAL SCORE	9	Conclusion: Score is ≤10. Unlikely to be a reverse innovation.

The first program, the Uganda Health Information Network (UHIN), provides community health workers with an online information system and personal digital assistants for recording health information [24]. The Bloomberg Philanthropies Maternal Health Initiative (BPMHI), on the other hand, trains health workers in performing life-saving procedures in maternal health in rural and remote areas of Tanzania [25].

UHIN has proven to be effective at improving health workers’ capabilities to access information remotely through a handheld digital device, leading to a score of 4 in Accessibility. It also has a proven ability to scale, having expanded successfully to 175 health facilities, therefore scoring 4 on Scalability. The program has demonstrated impact through an independent evaluation, which reported a 25% savings per patient visit unit through the new information system, compared to the original paper-based system, leading to a 5 on Cost Effectiveness. Thus, it has demonstrated some impact in this area, and it is in the process of evaluating its organizational performance and health system outcomes, leading to a 3 for Effectiveness.

Moving to Step 2, UHIN’s main mandate, health information integration and access to maternal healthcare, is highly relevant to the current Canadian policy environment, leading to a score of 5 on Gap in Target HIC. Given the general historical uncertainty of success associated with IT-based healthcare initiatives, however, UHIN scores a 3 in Compatibility with existing Canadian infrastructure. Mobile phone technology to record and store patient data in healthcare is relatively novel in the Canadian context, leading to a 4 on Novelty. Finally, UHIN is well-connected to Canadian partners, the International Development Research Centre and the former Canadian International Development Agency, leading to a 4 in Receptivity. This leads to an overall score of 16, with the conclusion that the program is likely to be worth testing as a reverse innovation in Canada.

Similarly, the Bloomberg Philanthropies Maternal Health Initiative scores well in Step 1. The initiative has improved access to healthcare for patients by upgrading remote health centers while expanding the skill set of midwives to include Cesarean sections, ostensibly improving the access to this much-needed procedure, and others, in rural areas, leading to a 5 on Accessibility. Through task shifting, the initiative has lower costs compared to staffing with physicians or long distance ambulance transportation, leading to a 4 on Cost-Effectiveness and Efficiency. The program has also demonstrated its scalability through its expansion from 9 to 12 sites, giving it a 4 on Scalability. At the same time, unlike the first initiative’s independent evaluation, BPMHI lacks specific data that measures the program’s impact, leading to a score of 2 on Effectiveness.

However, through Step 2, we find that UHIN has a much higher likelihood of success as a reverse innovation compared to BPMHI. The initiative certainly addresses an important gap in Canada, the accessibility of maternal healthcare in remote rural areas, scoring 4 on Gap in Target HIC, and uses a novel approach, scoring 3 on Novelty. Despite these positives, current Canadian regulation on scope of practice precludes midwives from doing surgery, leading to a rating of 1 on Compatibility. The program also lacks connections with existing Canadian partners, giving it a 1 on Receptivity. Given its overall rating of 9, the Bloomberg Philanthropies Maternal Health Initiative does not pass final round screening, and thus would not be further considered for further testing and adaptation into Canada.

## Discussion

The increasing pace of innovation in LMICs and the rising interest in cost control in HICs suggests that the value of systematically screening promising ideas from one setting for implementation in the other will rise over time. This paper describes a screening tool to rapidly assess promising LMIC innovations for adaptation in HICs and identify those with high potential for more in-depth review and evaluation. It highlights (but does not resolve) the tradeoff between high-potential radical innovations, which are difficult to implement, and incremental innovations that provide “easy wins.” It also highlights challenges in adapting LMIC innovations to HICs, including differences in scope of practice, quality concerns, and regulatory issues. It was developed with a broad range of experts from different sectors, and field tested for relevance to products, organizational processes, and policies. While our tool does not solve the challenge of adaptation from a low to high resource setting, it can help decide whether an approach is worth pursuing despite the barriers. It does so by including criteria for impact, cost, access, technical feasibility, and alignment with public policy.

Screening for reverse innovations may be useful for public, private, for-profit, and nonprofit organizations, particularly those that are interested in affordable approaches to meet the needs of marginalized or hard to reach groups. We envision that the metrics incorporated in the tool may be useful to a variety of stakeholders, including: multinational corporations, such as the Innovation Managers at Xerox who canvass Indian startups for innovations that may be implemented in their global operations [26]; health-care institutions, such as the Commonwealth Fund’s Innovation Collaborative, which brings together large health systems seeking novel approaches to increase efficiency; and governmental bodies, such as the New York City Council, which adapted Mexico’s conditional cash transfer program. For these stakeholders, the process of reverse innovation could be accelerated by using the criteria to screen databases for low-cost, high-yield innovations like the over 1300 programs listed in the CHMI database [22], the over 600 groups identified by Grand Challenges Canada [27], or the almost 100 examples in the World Innovation Summit for Health Innovation Network database [28]. Having a standard screening tool can help to proactively obtain the appropriate information from potential innovations and inform the further development of these databases. To this end, these criteria were tested for inter-rater reliability using a detailed dataset on innovations compiled by Imperial College in the United Kingdom for consideration by US delivery systems.

The strengths of this study include its multidisciplinary perspective, and its iterative development using numerous case examples, which contrast with the more ad hoc nature of previous work. The limitations include the narrow focus on application to the Canadian context, which may limit generalizability to other settings, the use of abstract concepts like compatibility, which may be subjective and limit agreement on how to assess each parameter, and finally, the limited data on the programs under study. The scores for each category are based on how strongly a program has demonstrated achievement in each area. While the scoring system and the cut-off scores were developed through several rounds of testing and feedback, there is some subjectivity in determining the scores for programs. The tool can be strengthened through further testing and standardization, which is currently ongoing at Imperial College. Next steps in this area would include testing in a country context other than Canada, and prospectively assessing the success of implementing innovations which scored higher with this tool with others having lower scores. The work of the Innovation Collaborative in the US, which has chosen 3 innovations to implement, can help to further this area of research, but more examples will be needed to assess the validity of the tool.

Summary screening of potential innovations is an important first step, but replication also requires identifying the key ingredients or efficiency core of an intervention, a process requiring more in-depth investigation [29]. CHMI has a tool to assist with this, looking at what makes the intervention work and its key contextual factors [30]. The University of California at Los Angeles’ Global Lab for Innovation has also drafted elaborate criteria to evaluate cost-saving innovations from across the globe with the aim of identifying and piloting these innovations within their health system [31]. Together, these tools help map the first steps in a complex process that draws on the ingenuity of people in low-resource settings as a source of new ideas for high-income settings, increasing the likelihood that they could have an impact.

## Conclusion

Diligent application of a tool to identify innovations from low-resource settings that improve affordability, access or quality could provide a range of options to improve the economics of healthcare in high-cost countries. As experience grows and more data becomes available, the tool can be refined and made more generalizable for the benefit of health organizations and decision makers worldwide. Once promising strategies are identified, the challenge of adapting innovations from LMICs to HICs remains, with opportunities for future research into the approaches that generate performance differences in translation itself. As the number of potential reverse innovations increase, a rapid screen should be followed by efforts to identify key components and contextual factors to increase their uptake and impact both in high- and low-income countries.

## Abbreviations

BPMHI: Bloomberg Philanthropies Maternal Health Initiative
CHMI: Center for Health Market Innovations
HIC: High- income countries
LMIC: Low- and middle-income countries
UHIN: Uganda Health Information Network

## Footnotes

1 We use the terms HIC and LMIC as described by the World Bank (https://datahelpdesk.worldbank.org/knowledgebase/articles/906519), which provides country categories based on gross national income (GNI) per capita. We make a distinction between HICs (those with a GNI per capita of $12,476 or more), and LMICs (those with a GNI per capita below $12,476).
